# Impact of screw design on refixation of solid avulsion fractures of the posterior cruciate ligament—A biomechanical feasibility study

**DOI:** 10.1002/jeo2.70225

**Published:** 2025-03-31

**Authors:** Thorben Briese, Christian Peez, Alina Albert, Arian Große‐Allermann, Elmar Herbst, Adrian Deichsel, Michael J. Raschke, Christoph Kittl

**Affiliations:** ^1^ Department of Trauma, Hand and Reconstructive Surgery University Hospital Muenster Muenster Germany

**Keywords:** avulsion fracture, headless compression screw, magnesium implant, posterior cruciate ligament, posterior drawer

## Abstract

**Purpose:**

Avulsion fractures of the posterior cruciate ligament (PCL) are commonly treated with refixation. Headless compression screws (HCS) offer benefits, compared to conventional techniques, such as reduced material irritation and option of bioabsorbable materials, possibly avoiding implant removal. Proofing its clinical applicability, the authors hypothesized that (1) biomechanical properties of HCS would be comparable to conventional techniques and (2) magnesium‐based HCS would provide comparable properties to titanium HCS.

**Study Design:**

Controlled laboratory study.

**Methods:**

Forty fresh frozen porcine knees were dissected keeping the menisci and ligaments intact. A solid avulsion fracture of the tibial PCL (20 mm [length] × 20 mm [width] × 10 mm [depth]) was created with a chisel. Specimens were randomized into four groups: (1) the native PCL, the tibial PCL avulsion refixed with parallel arranged, (2) two 3.5 mm cortical screws with washer (Ti‐CS), (3) two titanium headless compression screws (Ti‐HCS) or (4) two magnesium based headless compression screws (Mg‐HCS). Femur and tibia were mounted in a universal uniaxial testing machine (Zwick) simulating a posterior drawer testing, by performing axial load on the femur while the tibia was fixed in 90° flexion. After preconditioning, 500 cycles (200 mm/min) were performed with 10–100 N, followed by load‐to‐failure (LTF). Key parameters measured included stiffness, yield load, LTF and cyclic displacement. Differences were analyzed using an analysis of variance with a significance level of *p* < 0.05. The macroscopic failure mode was documented.

**Results:**

LTF of the intact PCL (1982.0 ± 285.4 N; *p* ≤ 0.001) significantly exceeded that of all refixations. Whereas LTF of Ti‐CS (1034.8 ± 236.1 N) significantly (*p* ≤ 0.01) exceeded those of HCS, no significance was seen between titanium (Ti‐HCS: 693.9 ± 220.5 N) or magnesium (Mg‐HCS: 686.7 ± 174.6 N) based HCS. No significant differences were found among the refixation methods regarding cyclic displacement or yield load. Stiffness for the Ti‐CS (144.0 ± 15.4 N/mm), Ti‐HCS (150.0 ± 22.9 N/mm) and Mg‐HCS (170.0 ± 20.9 N/mm) was lower than that of the intact PCL (190.9 ± 8.6 N/mm). Failure modes varied, with the Ti‐CS group showing PCL tears and the HCS groups always experiencing fragment luxation.

**Conclusion:**

In our biomechanical model, all fixation techniques demonstrated inferior biomechanical properties compared to the native PCL. Both HCS exhibited similar displacement, stiffness, and yield load values but showed a significantly lower LTF with fragment dislocation compared to conventional screws. Whether this difference is clinically relevant cannot be determined with this model as the porcine model only allows limited transfer into the clinical setting but might limit rehabilitation in the application of HCS in cases of solid avulsion fractures. Further clinical and in vivo studies should be followed to further differentiate the optimized fragment refixation technique.

**Level of Evidence:**

There is no level of evidence as this study was an experimental laboratory study.

**Abbreviations**
C: Celsius
Fig.: figure
HCS: headless compression screws
LTF: load‐to‐failure
MG‐HCS: magnesium‐based headless compression screws
min: minute
mm: millimetre
N: newton
PCL: posterior cruciate ligament
Tab.: table
Ti‐CS: titanium cortical screws
Ti‐HCS: titanium headless compression screws

## INTRODUCTION

The posterior cruciate ligament (PCL) serves as the primary stabilizer against posterior tibial translation [22, 23, 24]. PCL injuries typically result from posterior directed forces, most associated with motor vehicle or sports‐related accidents. While most PCL injuries are intrasubstance tears, avulsion fractures are relatively rare [15, 21]. Among these, tibial avulsions are more common than femoral avulsions [15, 21]. Surgical refixation is often necessary for PCL avulsion fractures to prevent complications [29, 33, 42]. Both open and arthroscopic techniques yield satisfactory results with low complication rates [17, 29] and surgical intervention generally leads to successful fracture healing and restoration of posterior tibial translation [15, 37].

The management of PCL avulsion fractures has evolved significantly over the years, leading to the development of various surgical techniques for refixation. These techniques range from open reduction and internal fixation using hooked plates or screws, to arthroscopically assisted procedures and suture bridge constructs, depending on the size and number of fragments involved [5, 6, 13, 16, 25, 44, 45, 47]. Recent biomechanical studies have analyzed various refixation techniques, including conventional screw osteosynthesis, suture bridge refixation, and arthroscopic suture button and pull‐out techniques [9, 12, 14, 27], seeking improved primary stability for accelerated rehabilitation. Although headless compression screws (HCS), may offer advantages in ligament repair such as less implant irritation, their biomechanical properties remain unknown. Furthermore, being available bioabsorbable (magnesium‐based), HCS might avoid the need for implant removal, providing benefits in revision surgeries.

Therefore, the aim of the present study was to compare the biomechanical properties (load‐to‐failure [LTF], displacement, yield load, stiffness and failure mode) of non‐ and bioabsorbable HCS screws with conventional screw osteosynthesis and to identify potential risk factors for clinical failure. We hypothesized that (1) the biomechanical properties of HCS would be comparable to those of conventional fixation methods. Additionally, we hypothesized that (2) bioabsorbable magnesium‐based HCS would provide comparable primary stability to titanium HCS.

## METHODS

Due to the use of cadaveric porcine specimens that were obtained from a local butcher, ethical approval was not required from the institutional review board of the University of Muenster. The porcine model was used as it is already well established in previous studies [9, 12, 14, 27] and provides comparability between results. Magnesium‐based HCS were provided by Medical Magnesium GmbH (Aachen, Germany). All other implants and materials were commercially purchased. Forty (*n* = 40) porcine cadaveric knee specimens were obtained from a local butcher and included in the study. Exclusion criteria encompassed any prior ligament or meniscus injuries, and ligamentous integrity was confirmed before testing. All 40 knees could be included in the final analysis. The specimens were stored at −20°C for a maximum of 4 weeks before testing and thawed at room temperature for 24 h prior to preparation and testing, which is consistent with most human cadaveric studies. The testing began when the specimens reached room temperature. To prevent tissue hysteresis, the knees were flexed 10 times before testing [30]. During dissection, the capsule, anterior extensor apparatus, and all associated muscles were removed, while the menisci, collateral ligaments and cruciate ligaments remained intact, which was adopted from previous studies [12, 13, 14, 45]. The tibia and femur were fixed in a steel pot using synthetic resin. Throughout testing, the specimens were kept moist with water to prevent dehydration. Adopted from our clinical practice, refixation was performed with two screws each.

### Study groups

The knees were manually randomized into four groups (each *n* = 10):

1. Native: native/intact PCL.
2. Ti‐CS: antegrade titanium cortical screw fixation (two 3.5 mm × 30 mm fully threaded + washer, Synthes).
3. Ti‐HCS: antegrade titanium headless compression screw fixation (two 3.5 mm × 30 mm partially threaded CCHS, Synthes).
4. Mg‐HCS: antegrade magnesium‐based headless compression screw fixation (two 3.5 mm × 30 mm partially threaded mm.CS, Medical Magnesium).

**Figure 1:**
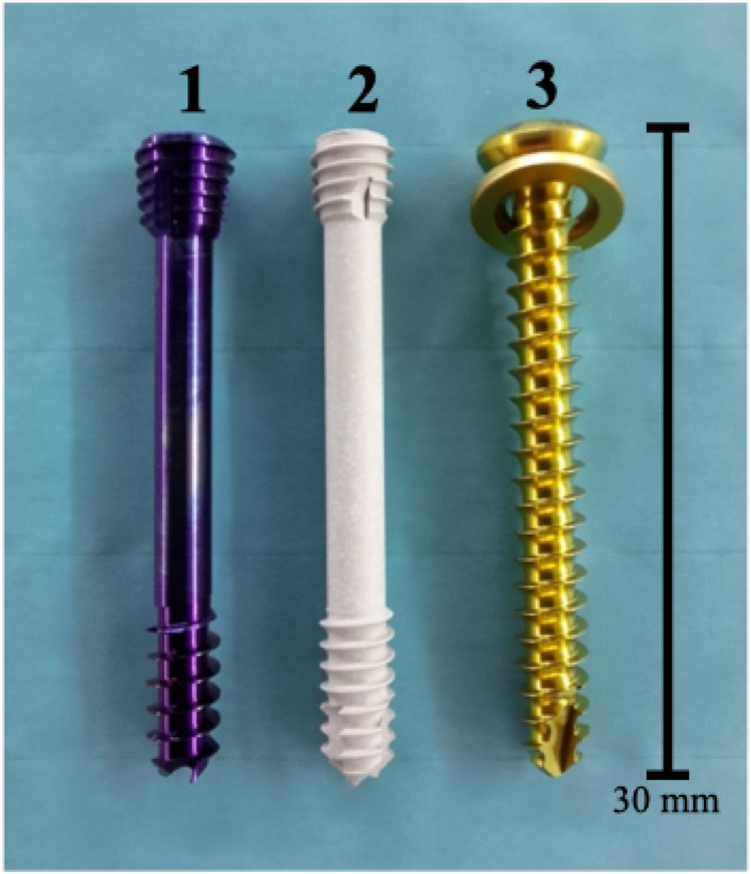
The used implants for refixation of the solid posterior cruciate avulsion Fragment, where differences between screw designs can be seen. (1) Titanium headless compression screw (3.5 mm × 30 mm, partially threaded CCHS, Synthes). (2) Magnesium‐based headless compression screw (3.5 mm × 30 mm, partially threaded mm.CS, Medical Magnesium). (3) Titanium cortical screw fixation (3.5 mm × 30 mm fully threaded + titanium washer, Synthes).

**Figure 2:**
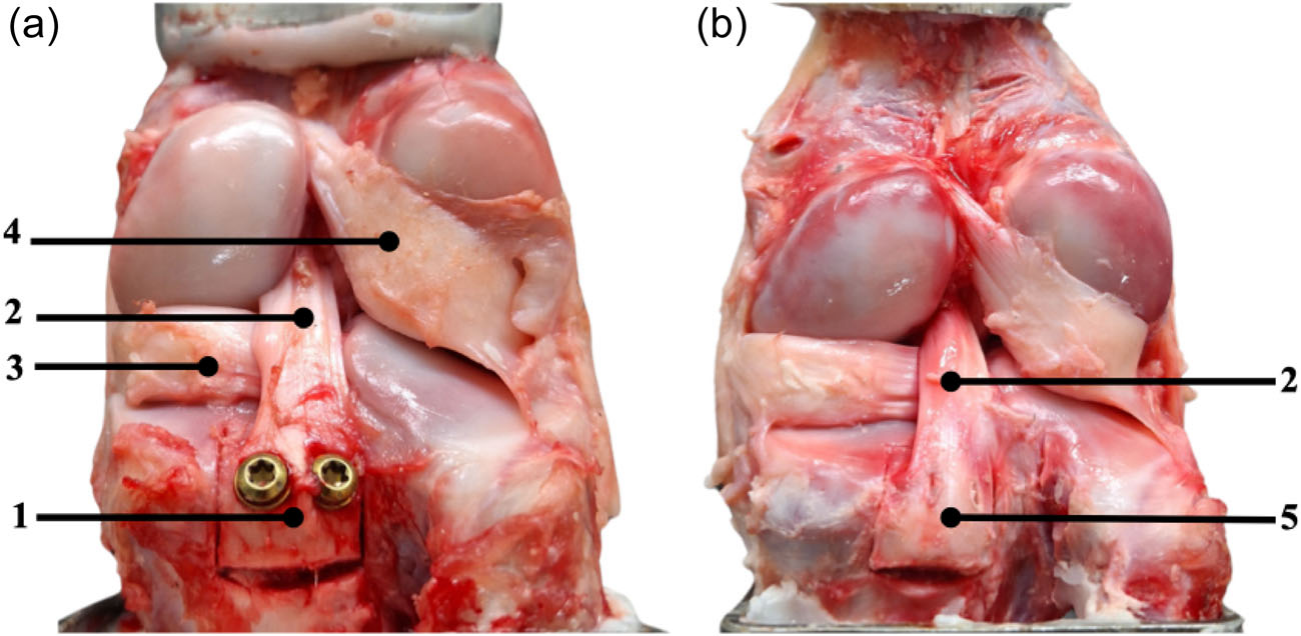
The specimens with refixed tibial avulsion fractures of the posterior cruciate ligament (PCL); (a) refixation of the avulsion fragment (1) was performed with two titanium fully threaded cortical screws with washer. It shows that the washer and the screw head lay above the PCL footprint. The PCL (2) itself was kept intact. The medial meniscus (3) and lateral meniscus (4) with meniscofemoral ligament were kept intact; (b) refixation of the avulsion fragment (5) was performed with two headless compression screws placed intracortical beneath the ligamentous tibial insertion. It shows that the screw head lays underneath the PCL footprint. The PCL (2) itself was kept intact.

The native specimen of the native group was tested with a complete and intact PCL, whereas a bony avulsion was performed for reconstruction. According to previous studies [9, 12, 27], a solid tibial fragment (20 mm [length] × 20 mm [width] × 10 mm [depth]) of the tibial insertion of the PCL was performed using a chisel. To simulate the fixation of Groups (2)–(4), reduction of the fragment in its fracture site was performed according to the manufacturer's information. Two parallel screws were used for refixation in each group according to our clinical practice and to provide more primary stability compared to single screw refixation. For the Ti‐CS group, two monocortical parallel 2.5 mm drill holes were created horizontally in the cranio‐caudal midline of the fragment, directed toward the tibial tuberosity according to previous studies [9, 12, 27]. Then the lag screw technique was performed with a 3.5 mm drill, allowing to fix the fragment with compression using two fully threaded screws (3.5 mm × 30 mm) with washers (Figures 1 and 2). In the Ti‐HCS group, two monocortical parallel 1.4 mm guide wires were placed horizontally in the cranio‐caudal midline of the fragment, also aimed at the tibial tuberosity. These guide wires were then over drilled with a 2.7 mm cannulated drill to create monocortical holes. Using the guide wires, two titanium cannulated HCS (3.5 mm × 30 mm) (Figures 1 and 2) were inserted to achieve compression. For the Mg‐HCS group, two monocortical parallel 1.1 mm guide wires were similarly positioned horizontally in the cranio‐caudal midline of the fragment, directed at the tibial tuberosity. These were over drilled with a 2.7 mm cannulated drill to create monocortical holes. Using the guide wires, two magnesium‐based cannulated HCS (3.5 mm × 30 mm) (Figures 1 and 2) were inserted to apply compression. The magnesium‐based screws are CE certified and manufactured from WE43MEO magnesium alloy and surface modified with plasma electrolytic oxidization (PEO). In Figure 2 the refixed defined tibial avulsion fragment of the PCL is shown, and differences between fixations can be seen.

### Biomechanical test setup

**Figure 3:**
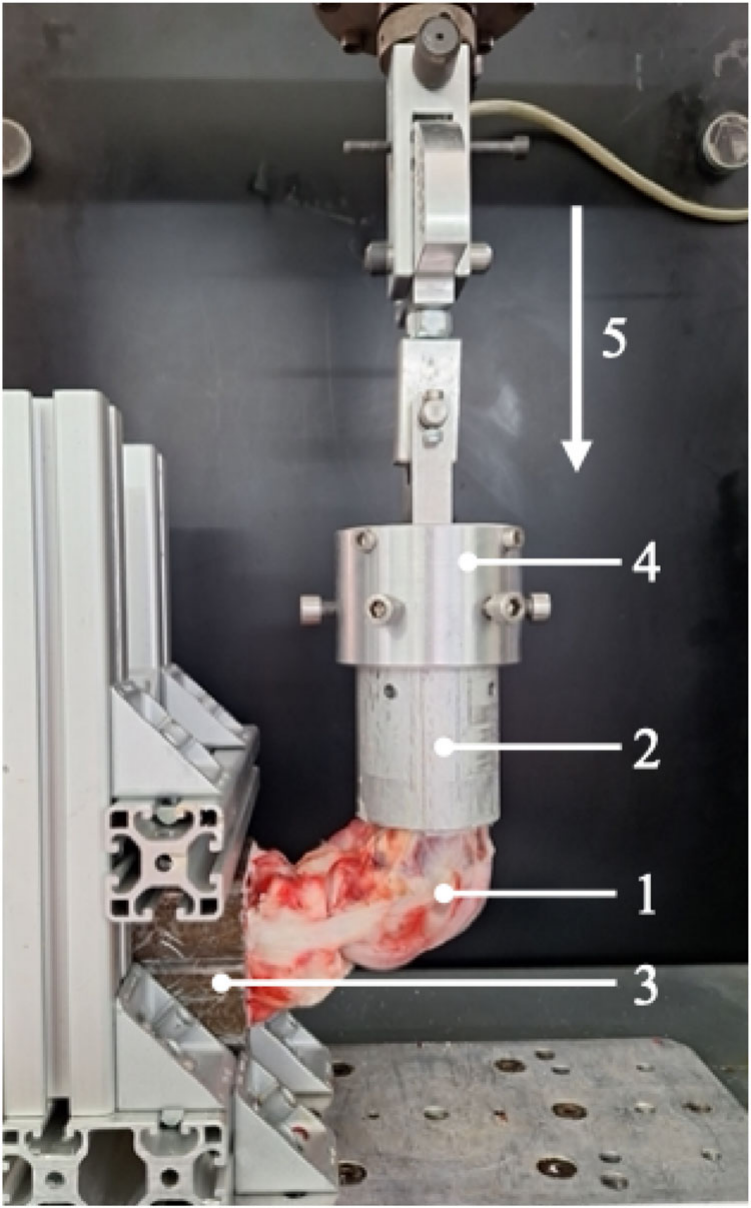
The testing setup with a left porcine knee specimen (1) mounted in the universal testing machine. The femur of the specimen is mounted in the steel pot (2) that is fixed to the custom‐made rig (4) of the universal testing machine. The tibia of the specimen is mounted in the tibial steel pot (3), which is mounted horizontally so that axial load (loading direction (5)) was performed on the femur in 90° flexion of the porcine knee joint simulating a posterior drawer test while the tibia is fixed.

Biomechanical testing was performed according to previous porcine models investigating refixation of tibial avulsion fractures of the PCL [9, 12, 14]. The femoral and tibial fixed specimens were placed in a universal uniaxial material testing system (Zwick/Roell) (Figure 3) that was utilized to determine the biomechanical primary stability. The knee was placed in 90° flexion. The tibia was fixed horizontally, and the femur was mounted vertically. The force was applied as an axial load on the femur creating a posterior drawer force. Preconditioning with 10 cycles with a load between 5 and 20 N was performed to minimize the viscoelastic effect. Then, cyclic loading with 500 cycles between 10 and 100 N with 200 mm/min was performed with subsequent LTF. For LTF, the construct was continuously loaded at a speed of 200 mm/min until failure of the construct occurred. Displacement (mm) after 100/200/300/400/500 cycles was analyzed, and LTF (N), stiffness (N/mm) and yield load (N) were calculated. Stiffness was calculated from the slope of the linear portion of the load–displacement curve during LTF. Yield load describes the strength leading to a plastic deformity. The macroscopic failure mode after the completed LTF was documented.

### Statistical analysis

Descriptive statistics, including mean and standard deviation, were calculated. Stiffness and yield load were calculated with a custom‐made Matlab (Version R2020a, MathWorks) script. The statistical analysis was performed using GraphPad Prism 10 (version 10.0.0). A one‐way analysis of variance (two‐way for comparison of cyclic displacement) was used for statistical analysis with Tukey's multiple comparisons test for post hoc correction. A *p* value less than 0.05 was deemed to identify significant differences. An a priori power analysis was performed using G*Power‐2 software (University Düsseldorf) [11]. A two‐tailed *t* test with means (difference between two independent means, two groups) was selected in G*Power‐2 for power analysis. Based on the means and standard deviations from a prior study [9], it was assumed that a sample size of 10 would allow for the identification of changes in LTF of 110 N, with a standard deviation of 80 N (effect size/Cohens' *d* = 1.4), with 80% power. Significance was set to a level of *p* < 0.05.

## RESULTS

### Displacement during cyclic loading

**Figure 4:**
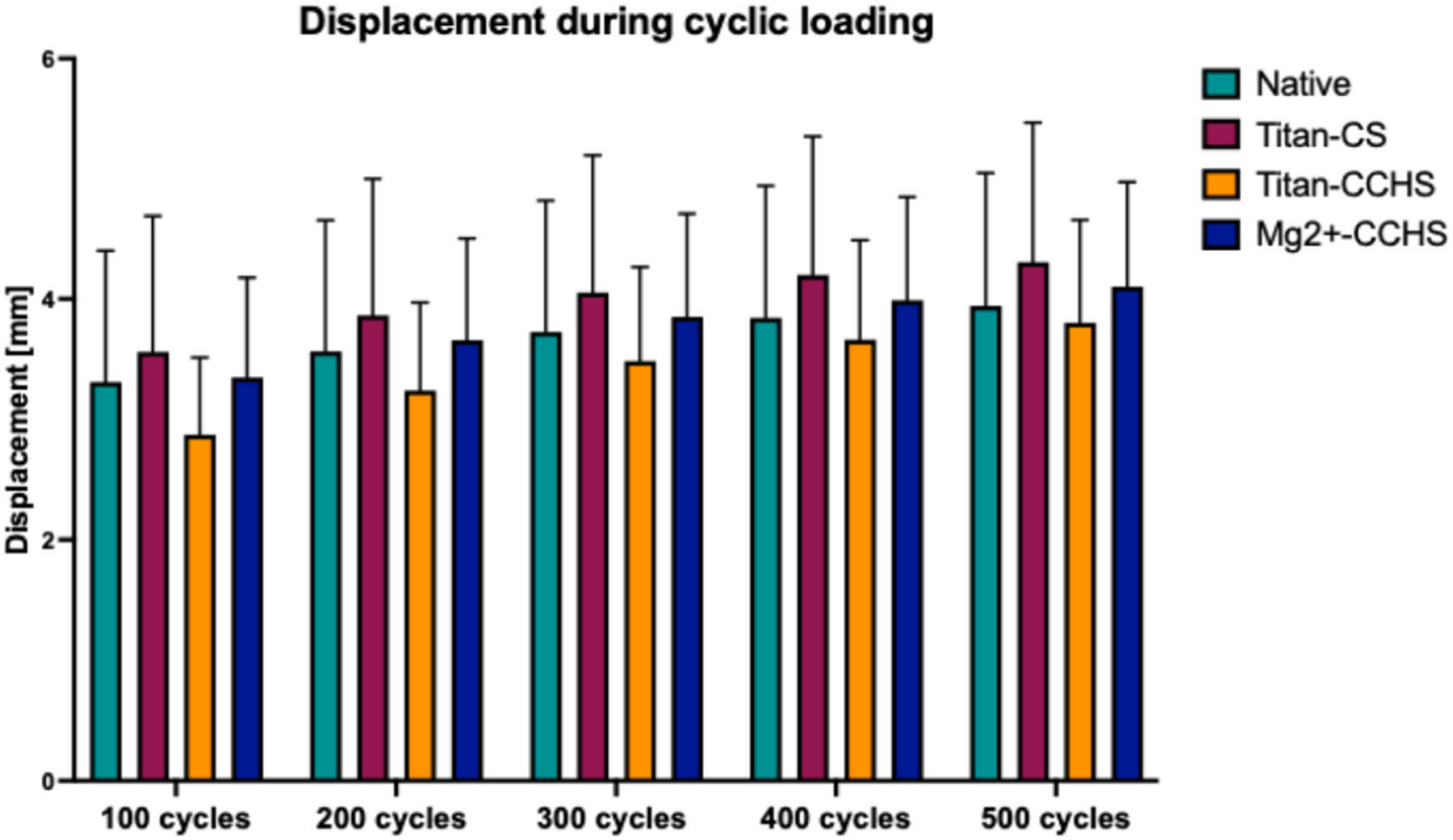
Displacement (mm) during cyclic loading after 100/200/300/400/500 cycles. No significant differences were shown between groups and cycles. Mg‐HCS, magnesium‐based headless compression screw; Ti‐CS, titanium cortical screw; Ti‐HCS, titanium headless compression screw.

During cyclic loading (Figure 4 and Table 1), all refixation techniques showed a continuous increase in displacement, whereas no significant differences between groups and cycles were shown.

**Table 1:** The displacement (mm) is presented as mean ± SD during cyclic loading after 100/200/300/400/500 cycles.

Cyclic displacement						
Cycles	100	200	300	400	500	*p*
Native	3.3 ± 1.1 (n.a.)	3.6 ± 1.1 (9.1%)	3.7 ± 1.1 (12.1%)	3.8 ± 1.1 (15.2%)	3.9 ± 1.1 (18.2%)	n.s.
Ti‐CS	3.5 ± 1.1 (n.a.)	3.8 ± 1.1 (8.6%)	4.0 ± 1.2 (14.3%)	4.1 ± 1.2 (17.1%)	4.2 ± 1.2 (20.0%)	n.s.
Ti‐HCS	2.9 ± 0.6 (n.a.)	3.2 ± 0.7 (10.3%)	3.5 ± 0.8 (20.7%)	3.7 ± 0.8 (27.6%)	3.8 ± 0.9 (31.0%)	n.s.
Mg‐HCS	3.3 ± 0.8 (n.a.)	3.7 ± 0.8 (12.1%)	3.9 ± 0.9 (18.2%)	4.0 ± 0.9 (21.2%)	4.1 ± 0.9 (24.2%)	n.s.

*Note*: Please see in brackets the percentage change to the loading after 100 cycles. No significance (n.s.) was observed between techniques and cycles. A one‐way ANOVA was performed to statistically analyze cyclic displacement in terms of groups and cycles.

Abbreviations: ANOVA, analysis of variance; Mg‐HCS, magnesium‐based headless compression screw; n.a., not applicable; Ti‐CS, titanium cortical screw; Ti‐HCS, titanium headless compression screw.

### LTF

**Figure 5:**
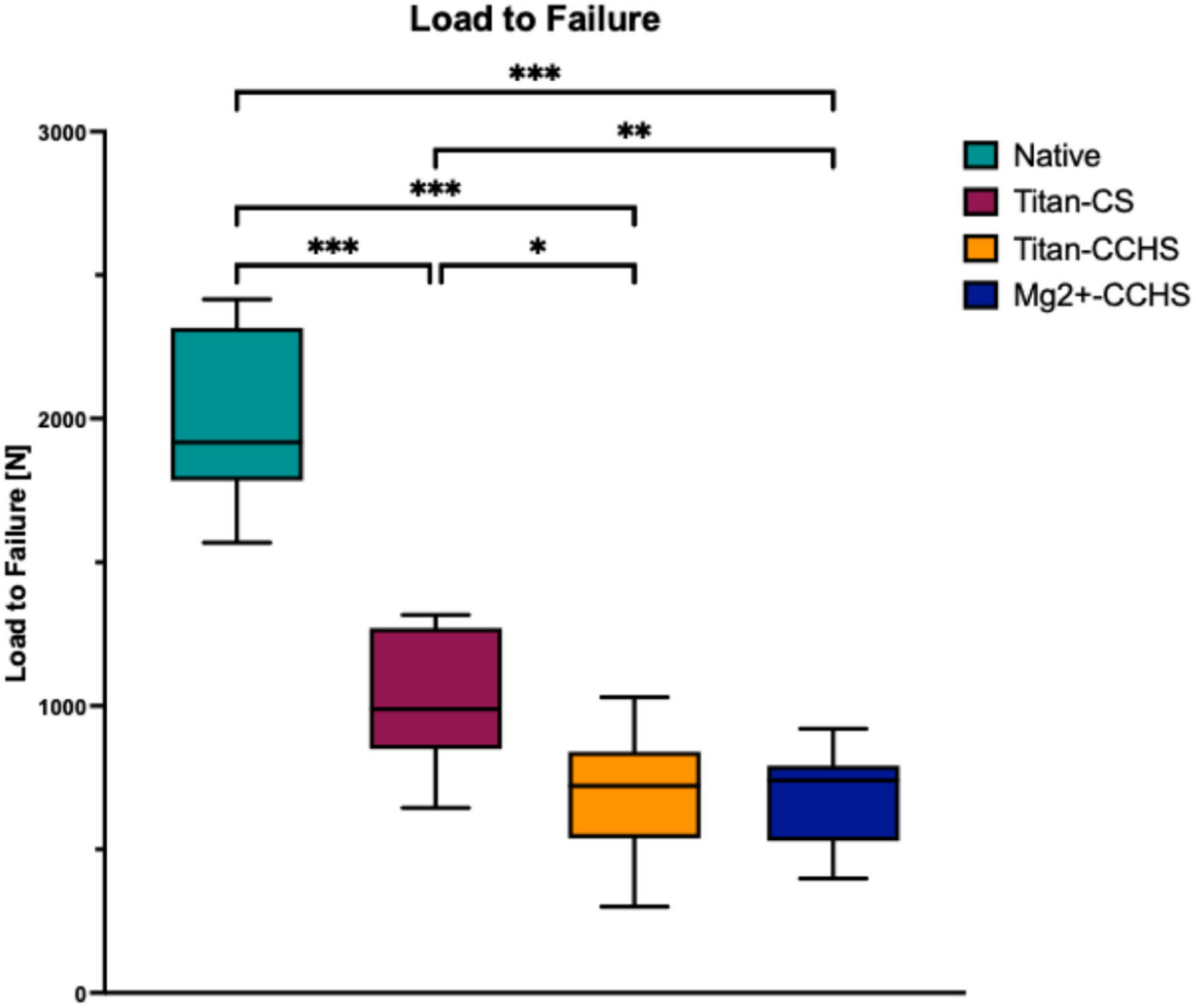
Load‐to‐failure in N. Mg‐HCS, magnesium‐based headless compression screw; Ti‐CS, titanium cortical screw; Ti‐HCS, titanium headless compression screw. Only significant differences are shown. *p ≤ 0.05; **p ≤ 0.01; ***p ≤ 0.001.

During LTF (Figure 5), all refixation techniques showed a significantly decreased LTF compared to the native state (1982.0 ± 285.4 N). Significant differences were seen between Ti‐CS (1034.8 ± 236.1 N) and Ti‐HCS (693.9 ± 220.5 N), and between Ti‐CS and Mg‐HCS (686.7 ± 174.6 N). No significant differences were seen between both HCS techniques.

### Stiffness

**Figure 6:**
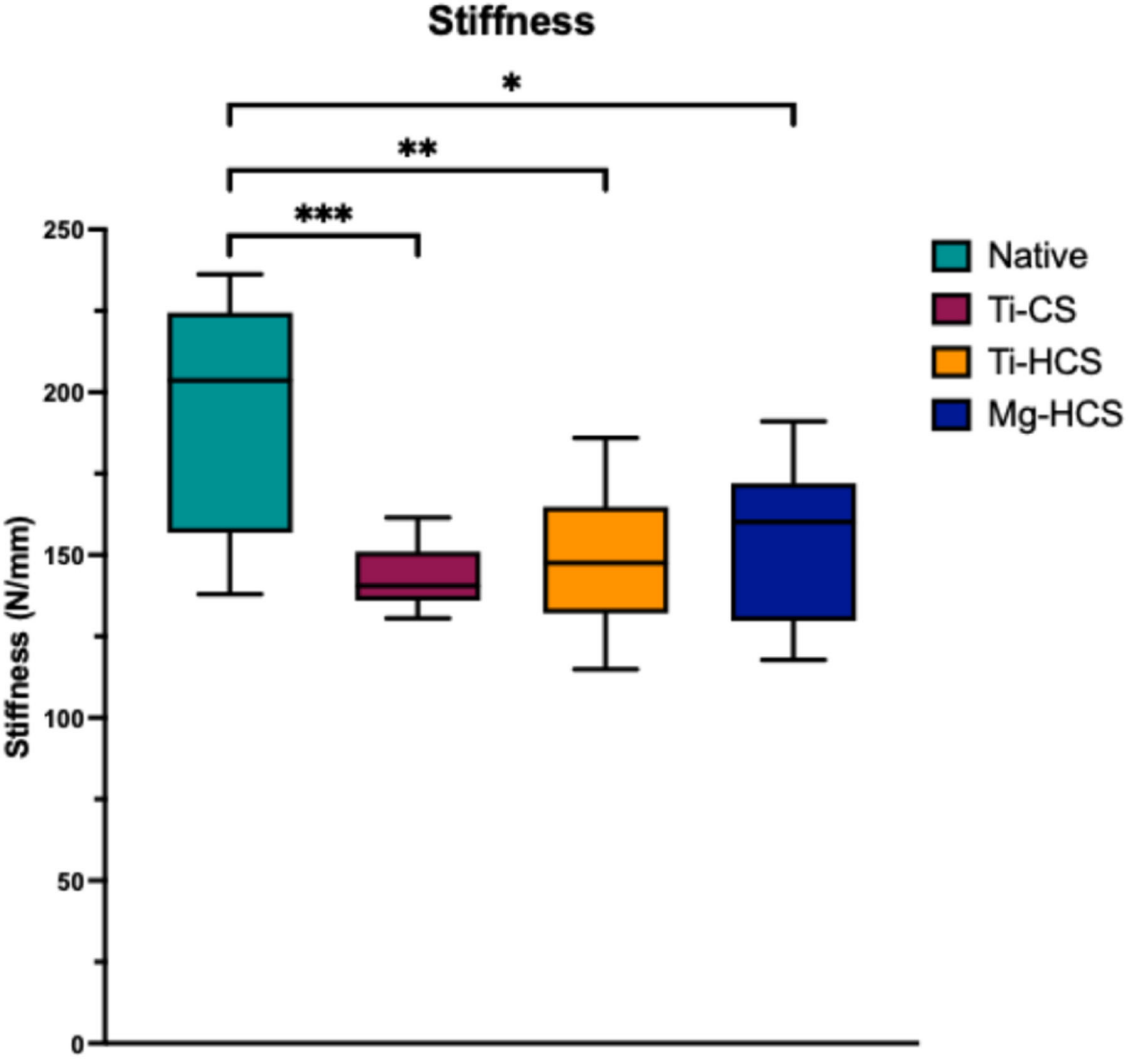
Stiffness in N/mm. Mg‐HCS, magnesium‐based headless compression screw; Ti‐CS, titanium cortical screw; Ti‐HCS, titanium headless compression screw. Only significant differences are shown. *p ≤ 0.05; **p ≤ 0.01; ***p ≤ 0.001.

Concerning the stiffness (Figure 6) of the different techniques, all refixation techniques showed a decreased stiffness compared to the native state (190.9 ± 8.6 N/mm). No significant differences were seen between all refixation techniques with Ti‐CS (144.0 ± 15.4 N/mm), Ti‐HCS (150.0 ± 22.9 N/mm) and Mg‐HCS (170.0 ± 20.9 N/mm).

### Yield load

**Figure 7:**
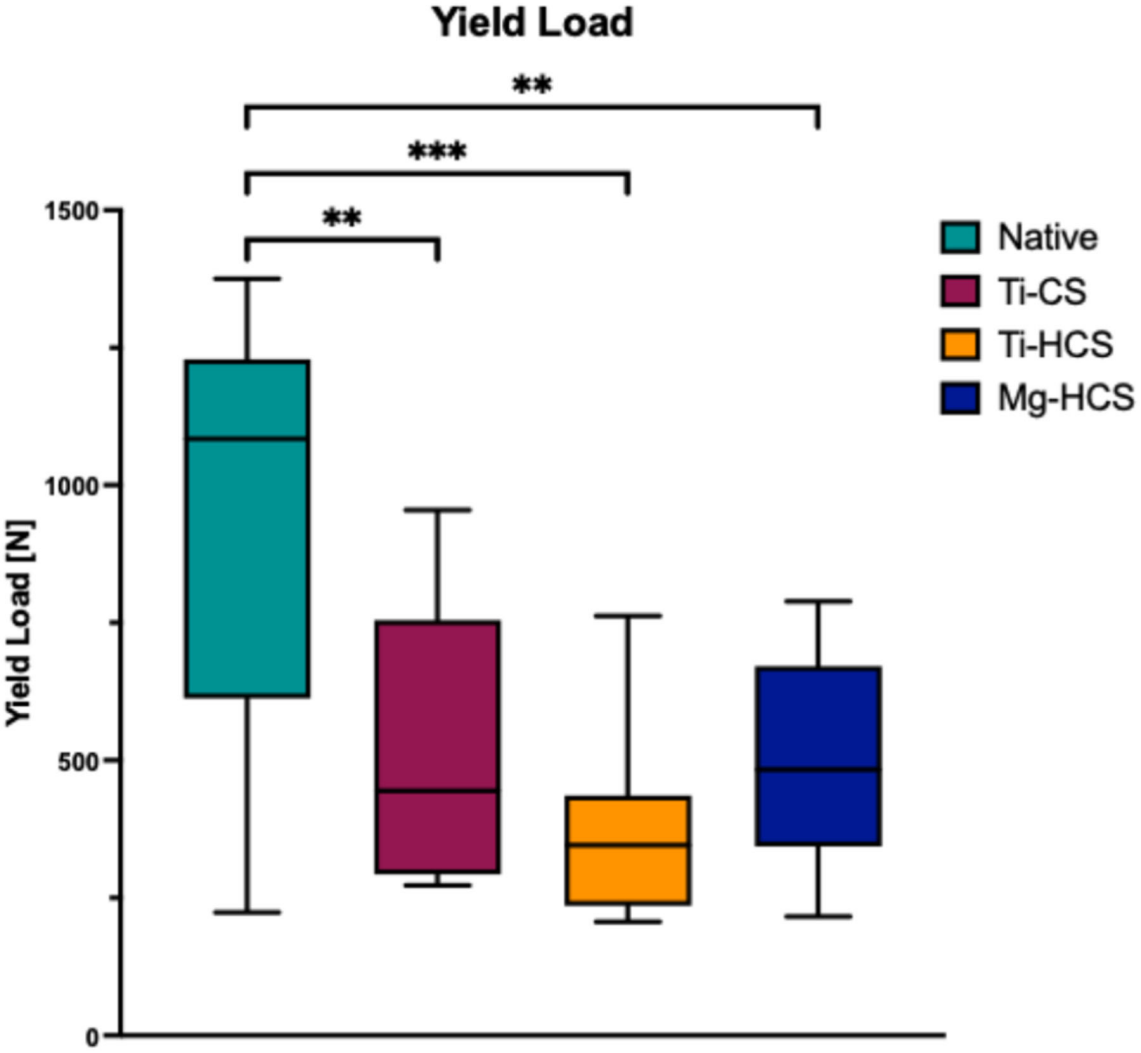
Yield load in N. Mg‐HCS, magnesium‐based headless compression screw; Ti‐CS, titanium cortical screw; Ti‐HCS, titanium headless compression screw. Only significant differences are shown. **p ≤ 0.01; ***p ≤ 0.001.

Concerning the yield load (Figure 7) of the different techniques, all refixation techniques showed a decreased yield load compared to the native state (941.3 ± 417.5 N). No significant differences were seen between all refixation techniques with Ti‐CS (524.6 ± 251.1 N), Ti‐HCS (367.7 ± 162.1 N) and Mg‐HCS (500.4 ± 193.1 N).

### Failure mode

Analyzing the failure mode (Table 2), we observed that in HCS groups, the failure mode was always an avulsion of the refixed fragment over the proximal partially threaded screw head, in all magnesium‐based and titanium HCS refixed specimens. In the native and Ti‐CS group we observed more heterogenous failure modes. In the native PCL group, we observed intrasubstance PCL tears in two specimens and eight avulsion fractures of the tibial ligamentous PCL insertion. In the Ti‐CS group, we observed nine PCL ruptures directly at the screw insertion site and 1 screw dislocation from its tibial screw insertion site.

**Table 2:** Failure mode and incidence of each failure mode in each group.

Group	*n*	Failure mode
Native	10	2× PCL intrasubstance tear (20%), 8× avulsion fracture of the tibial insertion due to open physis (80%)
Ti‐CS	10	9× PCL Rupture at screw insertion site (90%), 1× screw dislocation from tibial screw insertion (10%)
Ti‐HCS	10	10× Avulsion of fragment over the screw head (100%)
Mg‐HCS	10	10× Avulsion of fragment over the screw head (100%)

*Note*: 100% referring to *n* = 10 regarding each group.

Abbreviations: Mg‐HCS, magnesium‐based headless compression screw; PCL, posterior cruciate ligament; Ti‐CS, titanium cortical screw; Ti‐HCS, titanium headless compression screw.

## DISCUSSION

The most important finding of this study is, contrary to our hypothesis, that conventional screw osteosynthesis provided superior LTF values compared to HCS. However, all refixation methods demonstrated comparable biomechanical properties concerning stiffness, yield load and cyclic displacement. Whether these lower LTF values for HCS make a clinical difference cannot be answered with this study. Additionally, consistent with our hypothesis, our study shows that magnesium‐based HCS exhibit biomechanical primary stability comparable to that of contemporary titanium HCS. Furthermore, none of the repair techniques tested restored the structural properties of the intact PCL insertion in terms of LTF, displacement, yield load and stiffness. Importantly, no failures occurred during cyclic loading across any of the methods tested. In LTF testing for both titanium and magnesium‐based HCS groups, failure consistently manifested as fragment dislocation over the partially threaded screw head, rather than displacement or breakage of the HCS itself.

Previous studies have investigated the biomechanical properties of various refixation techniques for PCL avulsion fractures [9, 12, 14, 27]. However, the biomechanical properties of HCS for refixation of PCL avulsion fractures remain largely unexplored. In contrast to our findings, two previous studies [9, 12] with conventional screws showed much lower LTF values (681.5–817.5 N) compared to the present study. The reason for this may be that in these studies, only one screw (with and without) washer was used. Therefore, our findings indicate that using two cortical screws, might further enhance primary stability, which is the current practice in our clinic. Additionally, comparing these studies [9, 12] and our results, we can see that the application of a washer might enhance biomechanical properties. In previous studies, suture/suspension button refixation reached 286.8 N [14] to 842.0 N [27], whereas in our results, the HCS refixation reached 686.7–693.9 N. The Ti‐CS group achieved a stiffness of 144.0 N/mm, surpassing the stiffness of a single screw (63.3 N/mm [12] to 65.6 N/mm [9]) and suture/suspension button refixation (40.8–71.2 N/mm) [9, 12, 14, 27] reported in earlier studies. Interestingly, this is exceeded by our results of HCS refixation with 150.0–170.0 N/mm. Concerning cyclic displacement, after 500 cycles tested, we observed no significant differences compared to the native state or among the refixation techniques, with displacements ranging from 3.9 ± 1.1 mm (native) to a maximum of 4.2 ± 1.2 mm (Ti‐CS). However, it is important to mention that previous studies indicated significantly less displacement with suspension button constructs compared to screw fixation [9]. Concerning displacement, a recent clinical study showed that good and inadequately reduced PCL avulsion fractures led to comparable results concerning clinical results and patient‐reported outcomes measurements [48]. Nevertheless, even if the observed cyclic displacement is not significant, adopted and slow rehabilitation seem mandatory, especially as fragment luxation was observed in the HCS groups. Observing the failure modes, our study showed fragment luxation over the head of the HCS in HCS refixation. In contrast, different application angles of the HCS were not tested, which might modify fixation strength. We assume that in cortical screw refixation with a washer, the washer serves as a buttress providing compression and therefore avoiding fragment dislocation.

The clinical relevance of this study lies in testing the biomechanical feasibility of using HCS, particularly bioabsorbable HCS, for the refixation of PCL avulsion fractures. The peak strain on the PCL during daily activity and rehabilitation is reported to be very inhomogeneous with different approaches [10, 18]. This includes ground walking with up to 0–160 N [18, 38, 40, 46] or 0.2–1 times body weight [3, 18, 19, 39], respectively. Furthermore, seated active extension reaches up to 74 N [43], active flexion can reach up to 3330 N [43] and common rehabilitation exercises like leg presses or squats/lunges up to 2700 [10, 43] PCL tensile forces. However, PCL tensile forces during extension and walking were exceeded by all our tested refixation techniques; no technique reached the PCL tensile forces applicable during progressive rehabilitation. Therefore, refixation of displaced PCL avulsion fractures should include partial weight bearing and controlled rehabilitation. Our findings suggest that displaced PCL avulsion fractures may also be effectively treated with HCS, but conventional screw osteosynthesis with two screws remains superior over HCS and comparable suture‐based refixation techniques. This could impact patient outcomes when using HCS, as progressive rehabilitation should be more restricted due to their lower LTF compared to refixation with cortical screws. Therefore, HCS might not be suitable if progressive rehabilitation is aimed or if patients present with an increased body weight, which could increase loading on the refixation. Additionally, due to the risk of fragment dislocation around the head of the HCS, multi‐fragment avulsion fractures may not be suitable for HCS refixation. Furthermore, bioabsorbable magnesium‐based HCS also exhibit biomechanical performance similar to that of titanium HCS. However, clinical studies assessing HCS refixation for PCL avulsion fractures are still missing. Due to their headless design, both Ti‐HCS and Mg‐HCS may cause less material irritation in cases where open refixation of solid PCL avulsion fractures is performed. Additionally, the bioabsorbable nature of Mg‐HCS can eliminate the need for implant removal, which could be advantageous in scenarios where secondary PCL reconstruction is required due to recurrent instability. Considering our results, refixation using Ti‐HCS and Mg‐HCS appears safe for the treatment of PCL avulsion fractures, particularly when weight‐bearing restrictions are implemented during the fracture healing process. Furthermore, it should be considered that the traditional technique with cortical screws may be more cost‐effective, as HCS implants are more expensive, as well as suture‐button or suture‐bridge constructs. However, this cost difference might be negligible, since magnesium‐based HCS implants do not need to be removed. However, PCL avulsion fractures are relatively rare [15, 21], and therefore our findings are only relevant to a small and specific subset of patients.

This study had several limitations that must be considered before the application of our results into clinical practice, which are inherent to in‐vitro biomechanical studies. Previous studies have shown the suitability of the porcine knee for use in biomechanical investigations [35], but bone density in the porcine model is higher in comparison with the human knee joint [1]. Additionally, porcine anatomy and the slope of the porcine knee are different compared to human knee specimens, which might alter the posterior tibial translation in the applied biomechanical test setup. Furthermore, biomechanical testing was a simulation of forces acting at time zero, and biological factors and fracture healing of the PCL avulsion fracture, as well as degradation of the Mg‐HCS, was not considered, which might have influenced especially the relevance of the tested cyclic displacement. Additionally, due to possible degradation and osteolysis of magnesium‐based implants, long‐term outcomes cannot be predicted. Therefore, possible influences of the different materials on bone healing cannot be excluded, as in vivo testing might alter the results. Nevertheless, the porcine model has already been evaluated and proven in previous biomechanical studies investigating refixation techniques of PCL avulsion fractures [9, 12, 14, 27]. Due to the testing protocol, preparation of the capsule and extensor apparatus was necessary, which might alter the kinematic of the native knee joint and increase the forces acting on the reconstruction. In addition, different angles of screw placement, which may affect primary stability, were not analyzed. Furthermore, different screw designs might alter the primary stability as well. Despite advancements in new‐generation magnesium‐based implants that control degradation and reduce gas production during oxidation [2, 7, 20, 26, 28, 31, 36], surgeons should remain aware of potential complications such as unpredictable degradation and intraosseous osteolysis [28, 32, 34, 41] which could lead to secondary complications. Nevertheless, radiolucent zones during the degradation of magnesium‐based implants are often a self‐limiting phenomenon [8]. Furthermore, the displacement was measured indirectly via the crossbar of the universal uniaxial material testing system rather than direct fragment dislocation. This may have overestimated the dislocation, as previously shown [4]. Potential modifications in this biomechanical model with directly monitored fragment dislocation, human cadaveric studies, and in vivo and clinical studies are possible future directions for identifying the optimal refixation technique.

## CONCLUSION

In our biomechanical model, all fixation techniques demonstrated inferior biomechanical properties compared to the native PCL. Both headless compression screws exhibited similar displacement, stiffness and yield load values but showed a significantly lower LTF with fragment dislocation compared to conventional screws. Whether this difference is clinically relevant cannot be determined with this model as the porcine model only allows limited transfer into the clinical setting but might limit rehabilitation in the application of HCS in cases of solid avulsion fractures. Further clinical and in vivo studies should follow to further differentiate the optimized fragment refixation technique.

## AUTHOR CONTRIBUTIONS

Conception and design, testing and data acquisition, statistical analysis and writing: Thorben Briese and Christoph Kittl. Internal review and statistical analysis: Christian Peez and Alina Albert. Internal review, testing and data acquisition: Arian Große‐Allermann. Internal review: Elmar Herbst, Adrian Deichsel and Michael J. Raschke.

## CONFLICT OF INTEREST STATEMENT

Elmar Herbst is Deputy Editor‐in‐Chief for the *Knee Surgery, Sports Traumatology and Arthroscopy (KSSTA)*. Adrian Deichsel is Web Editor for the *Knee Surgery, Sports Traumatology and Arthroscopy (KSSTA)*. The remaining authors declare no conflicts of interest.

## ETHICS STATEMENT

Ethical approval from the Institutional Review Board of the University of Münster was not required for this study, as porcine specimens were used.

## Data Availability

Data are available from the corresponding author upon reasonable request.
